# Severe gestational hypertriglyceridemia: a rare but serious situation

**DOI:** 10.11604/pamj.2019.34.13.20065

**Published:** 2019-09-06

**Authors:** Anissa Ben Amor, Kaouther Dimassi, Moez Attia, Chiraz Amrouche, Amel Triki

**Affiliations:** 1 University Tunis El Manar, Faculty of Medicine of Tunis, Tunis, Tunisia; 2 Department of Obstetrics and Gynecology, Mongi Slim Hospital, Tunis, Tunisia; 3 Unit A, National Nutrition Institute, Tunis, Tunisia

**Keywords:** Hypertriglyceridemia, pregnancy, acute pancreatitis, management

## Abstract

The severe hypertriglyceridemia during pregnancy is a rare condition. It is a problem for diagnostic, prognostic and therapeutic. This dyslipidemia benefit from specific and effective treatments, but it is still poorly codified. Dietary is still the essential therapeutic, but fetal extraction should also be considered if the gestational age permits. Post-partum monitoring is required but etiologic thorough is not recommended if the triglycerides rate normalizes. The major complication of hypertriglyceridemia should be actively sought because of important maternal mortality rate.

## Introduction

During pregnancy, plasma triglyceride levels normally increase, and this is usually of little clinical consequence. Hypertriglyceridemia is more pronounced during the 2^nd^ and 3^rd^ trimester of pregnancy [1, 2]. Severe hypertriglyceridemia in pregnancy is a rare condition and usually occurs in the third trimester [3]. It is often multifactorial and threatens maternal and fetal prognosis by increasing incidence of complications such as preeclampsia or fetal macrosomia [2-4] and exposing the mother to its major complication: acute pancreatitis [5, 6]. It justifies urgent care based primarily on dietary measures and fetal extraction if the term permits. Most of the cases of severe gestational hypertriglyceridemia that have been reported previously in the literature were caused by genetic mutations or familial hypertriglyceridemia [7]. We report a case of severe, non-genetic, non-familial, pregnancy-induced hypertriglyceridemia. We report the management modalities of this disease and maternal-fetal complications.

## Patient and observation

**Figure 1:**
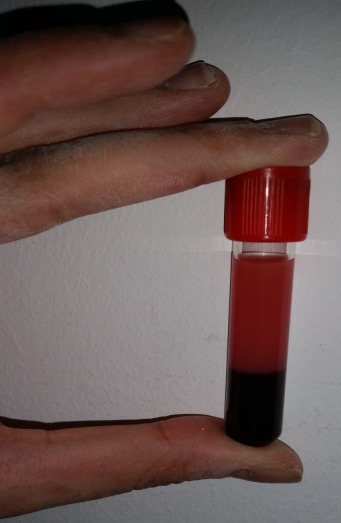
Patients plasma aspect

**Figure 2:**
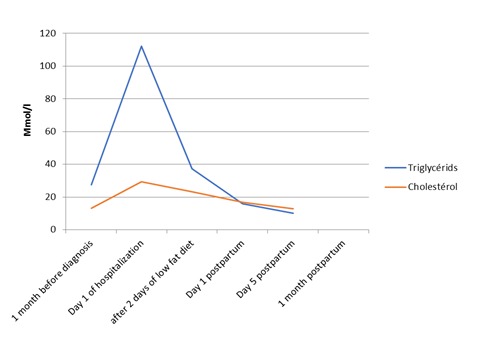
Changes of the triglycerids and cholesterol plasma level

A 34-year-old woman, with no medical history, presented to the hospital in her third pregnancy, at 30 weeks of amenorrhea, for hyperemesis. Her previous pregnancy, was uneventful and she vaginally delivered a healthy child. The current pregnancy was a monochorionic diamniotic twin pregnancy, irregularly followed but uneventfully progressed and all her antenatal screening tests were normal. Her diabetes screening was negative. At the first physical examination, the patient had a blood pressure at 160/90 mmHg, with 2 cross proteinuria at the strip test without other signs of pre-eclampsia and normal deep tendon reflexes. Her abdomen was supple, but she has irregular uterine contractions. Her cervix was 2 cm dilated with intact membranes. Both fetuses monitoring were normal with normal growth at ultrasound examination. The patient was admitted in units of pathological pregnancies care for suspected preeclampsia in preterm labor context. A full blood count taken, to check her vascular and renal function, was noted to be grossly lipemic (Figure 1). A fasting blood specimen showed elevated cholesterol and triglyceride. A severe hypertriglyceridemia to 108,3 mmol/l (normal value <2, 28 mmol/l) and a severe hypercholesterolemia 28,38 mmol/l (normal value <5.16 mmol/l) were found. Serum analysis revealed normal glucose, amylase, lipase and thyroid stimulating hormone. Urea, electrolytes and liver function tests were all normal. The 24-hours proteinuria was positive (0,4 g/24h). Abdominal ultrasound scan was unremarkable with no evidence of cholelithiasis, a normal pancreas and no hepato-splenomegaly. The patient was put under calcium channel blockers intravenously as tocolysis and antihypertensive. Corticotherapy for fetal lung maturation has been started. The blood pressure was stabilized and the uterine contractions have stopped initially. The patient was affected by a type V hypertriglyceridemia, according to the Fredrickson/WHO classification of hyperlipoproteinemia. This subtype is characterized by the presence of severe hypertriglyceridemia associated with hypercholesterolemia. Acute pancreatitis was eliminated by the clinic, biology and abdominal ultrasound. The hyperlipidemia was controlled by an extremely low-fat diet. The patient was managed initially by intravenous fluids with a low-carbohydrate. The plasma lipid levels quickly decreased with this diet. Fetal extraction was performed by caesarean section at 31 WA, due to secondary persisting uterine contractions despite the tocolysis. The patient delivered two male new born, weighing 1900g and 2050g. They were supported by the neonatology team. The evolution was favorable during postpartum with a normalization of blood pressure, a negativity of proteinuria and good regression of triglycerides and cholesterol plasma levels (Figure 2). The patient was then supported by endocrinologists for further investigations and monitoring. After two months of following, the lipid plasma levels still normal and there were no abnormalities in other explorations. Both newborns showed a neonatal severe hypertension requiring antihypertensive intravenous treatment. This hypertension remains unexplained despite all the explorations.

## Discussion

The increased plasma lipids levels during pregnancy aim to meet fetal needs. During normal gestation, serum cholesterol and triglycerides increase progressively, particularly in the third trimester, by 25-50% and 200-300%, respectively [1]. In most cases, the severe hypertriglyceridemia in pregnancy occurs on pre-existing idiopathic, genetic, or ethylic dyslipidemia [3, 8]. In the absence of predisposing factors, it is often associated with diabetes, even gestational [8]. Our observation is even a rarer situation where severe hypertriglyceridemia is revealed by pregnancy but with no predisposing factor to explain it. Whatever field occurrence, the hypertriglyceridemia threatens maternal and fetal prognosis by increasing incidence of complications such as preeclampsia [2], or fetal macrosomia (independently of other factors) [1] and exposing to acute pancreatitis (AP) (4 to 6% of AP during pregnancy are linked to a hypertriglyceridemia [6]. Precisely, our patient had a preeclampsia. The risk for acute pancreatitis increased when plasma triglyceride levels are >1000 mg/dl (11.3 mmol/l). However, as in this case, some patients do not develop acute pancreatitis even with very high serum triglyceride level [5, 9]. Commonly, acute pancreatitis occurs in women with pre-existing hypertriglyceridemia. Furthermore, familial hypertriglyceridemia is often complicated with acute pancreatitis in pregnancy [9]. The diagnosis may not be confirmed by detecting increased serum amylase activity, as artefactually low activity may be secondary to interference by triglyceride-rich lipoproteins in the laboratory assay [6]. The risk of maternal and fetal mortality because of gestational pancreatitis are approximately 15-20% each [9]. In our case, we tried to eliminate the pancreatitis diagnosis by the clinic, testing the lipasemia, performing abdominal ultrasound and regular serum amylase assay.

Further, the hypertriglyceridemia is a controversial risk factor for coronary heart disease. Therefore, it is difficult to make a precise estimate of the long-term risk [1, 4, 8]. However, there is evidence to suggest that, during pregnancy, maternal hypercholesterolemia is associated with the development of fetal atherosclerosis, but its clinical significance is still unknown. In this case, both newborns had severe hypertension that was unexplained, but there are no other reports of similar cases. Our patient responded very well to the dietary regimen alone (Figure 2). No lipid regulating drugs were needed in our case. The basic treatment of gestational hypertriglyceridemia is based on a low-fat diet (less than 10-20 % of daily calories depending on the severity). If it is not enough, statins are the drug of choice in treating hypercholesterolemia along with fibrates such as gemfibrozil for treating combined hypercholesterolemia and hypertriglyceridemia. Some teams offer additional treatment with omega-3 polyunsaturated fatty acids in capsule [3]. Most fibrate drug information sheets and formularies, state that fibrates are contraindicated in pregnancy, there have been a few reports of the successful use of gemfibrozil, to reduce the risk of developing hypertriglyceridemic pancreatitis during pregnancy with no side effects particularly in the third trimester. Furthermore, improving glycemic control could decrease triglyceride levels in diabetes-associated hypertriglyceridemia. Insulin could be used in these cases [1]. Pregnant women with hypertriglyceridemia should use low fat diet to keep triglyceride levels below 885 mg/dl (10 mmol/l). Furthermore, the same treatment could be used in subsequent pregnancies to prevent gestational hypertriglyceridemia. Other treatment options in resistant cases include plasma exchange, plasmapheresis, IV heparin and total parenteral nutrition [3, 4].

If the hypertriglyceridemia is major and complicated, quickly effective treatment should be initiated urgently. It is based in priority on the interruption of pregnancy if the gestational age permits, often leading to a rapid improvement of lipid plasma levels (10-20% decrease in triglyceride levels within the next 24 h) and in parenteral nutrition without lipid. But timing of delivery in this condition is controversial [3, 4, 9]. The patient in this case underwent cesarean because of preterm labor and the delivery potentiated the lipid plasma levels regression (Figure 2). The effect of delivery on the decline of plasma triglyceride levels can be immediate and significant. However, the maternal and fetal condition should be taken into consideration especially in pre-term deliveries. Therefore, early delivery by induction of labor or elective caesarean section has been advocated particularly in cases with persistent hypertriglyceridemia to reduce the risk of developing acute hyperlipidemic pancreatitis with its high maternal and perinatal mortality [3, 4]. There is the question of contraception and the risk of recurrence in subsequent pregnancies. The oral contraception is forbidden because of the risk of major hypertriglyceridemia and AP. Contraception by copper intrauterine device is the best solution and only progestin contraception can be considered at minimum dose and under regular laboratory monitoring [3]. There is no indication for use of genetic etiological testing in routine in cases of hypertriglyceridemia [7]. However, the risk of recurrence in case of new pregnancy warrants close clinical and laboratory monitoring from the second trimester of pregnancy [4].

## Conclusion

The severe hypertriglyceridemia during pregnancy is a rare condition. It is a problem for diagnostic, prognostic and therapeutic. This dyslipidemia benefit from specific and effective treatments, but it is still poorly codified. Dietary is still the essential therapeutic, but fetal extraction should also be considered if the gestational age permits. Post-partum monitoring is required but etiologic thorough is not recommended if the triglycerides rate normalizes. The major complication of hypertriglyceridemia should be actively sought because of important maternal mortality rate.

## Competing interests

The authors declare no competing interests.
